# Recent advances in additive manufacturing of patient-specific devices for dental and maxillofacial rehabilitation

**DOI:** 10.1016/j.dental.2024.02.006

**Published:** 2024-02-24

**Authors:** Monireh Kouhi, Isaac J. de Souza Araújo, Farah Asa’ad, Lubna Zeenat, Sri Sai Ramya Bojedla, Falguni Pati, Ali Zolfagharian, David C. Watts, Marco C. Bottino, Mahdi Bodaghi

**Affiliations:** a Dental Materials Research Center, Dental Research Institute, School of Dentistry, Isfahan University of Medical Sciences, Isfahan 81746-73461, Iran; b Department of Cariology, Restorative Sciences, and Endodontics, University of Michigan, School of Dentistry, Ann Arbor, MI, United States; c Department of Biomaterials, Institute of Clinical Sciences, Sahlgrenska Academy, University of Gothenburg, Gothenburg, Sweden; d Department of Oral Biochemistry, Institute of Odontology, Sahlgrenska Academy, University of Gothenburg, Gothenburg, Sweden; e School of Engineering, Deakin University, Geelong 3216, Australia; f Department of Biomedical Engineering, IIT Hyderabad, Kandi, Sangareddy, Telangana 502285, India; g School of Medical Sciences, University of Manchester, Manchester, UK; h Department of Biomedical Engineering, College of Engineering, University of Michigan, Ann Arbor, MI, United States; i Department of Engineering, School of Science and Technology, Nottingham Trent University, Nottingham NG11 8NS, UK

**Keywords:** 3D printing, 4D printing, Patient-specific treatment, Image-based devices, Dentistry, Maxillofacial Regeneration

## Abstract

**Objectives:**

Customization and the production of patient-specific devices, tailoring the unique anatomy of each patient’s jaw and facial structures, are the new frontiers in dentistry and maxillofacial surgery. As a technological advancement, additive manufacturing has been applied to produce customized objects based on 3D computerized models. Therefore, this paper presents advances in additive manufacturing strategies for patient-specific devices in diverse dental specialties.

**Methods:**

This paper overviews current 3D printing techniques to fabricate dental and maxillofacial devices. Then, the most recent literature (2018–2023) available in scientific databases reporting advances in 3D-printed patient-specific devices for dental and maxillofacial applications is critically discussed, focusing on the major outcomes, material-related details, and potential clinical advantages.

**Results:**

The recent application of 3D-printed customized devices in oral prosthodontics, implantology and maxillofacial surgery, periodontics, orthodontics, and endodontics are presented. Moreover, the potential application of 4D printing as an advanced manufacturing technology and the challenges and future perspectives for additive manufacturing in the dental and maxillofacial area are reported.

**Significance:**

Additive manufacturing techniques have been designed to benefit several areas of dentistry, and the technologies, materials, and devices continue to be optimized. Image-based and accurately printed patient-specific devices to replace, repair, and regenerate dental and maxillofacial structures hold significant potential to maximize the standard of care in dentistry.

## 1. Introduction

Three-dimensional (3D) printing technologies, also known as additive manufacturing (AM) or rapid prototyping, have permeated every aspect of life, resulting in a great revolution in how products are designed, developed, and fabricated. In ISO/ASTM 52900(en) standard, 3D printing is defined as the layer-by-layer addition of materials to manufacture 3D constructs from a 3D model data[1]. The myriad of AM technologies has shown several advantages over subtractive manufacturing. These include controlling inner structure with high precision, saving materials, designing objects as single units, electronic file transfer without occupying physical space, and personalized customization [2,3]. The evolution of 3D printing in the biomedical field has followed the concept of personalized therapy, where the medical treatments are tailored to the patient’s characteristics, needs, and preferences, as well as their specific diagnosis, therapy, and follow-up [4,5].

In the dental field, 3D printing appliances are used in various specialties to fabricate patient-specific surgical guides, 3D models for pre-surgical planning in craniomaxillofacial surgery, dental implants, restorative and prosthetic devices, etc. [6,7]. There is also the utilization of 3D printing to produce tissue-engineered scaffolds and prostheses using biocompatible materials that perfectly fit the patient’s anatomical features as indicated by diagnostic imaging tools [8,9]. For instance, data collected using different extraoral or intraoral scanning instruments is processed to design virtual models using computer-aided design (CAD) software. Subsequently, a tessellation STereo-Lithography (STL) file is created and imported to the printer software. The technical parameters, variables, and materials are then determined to fabricate the patient-specific constructs [10]. Furthermore, recent progress in the 3D printing of advenced materials has led to the development of a new generation of “dimensional printing” known as four-dimensional (4D) printing. A 4D printing technology combines 3D printing with time as the fourth dimension [11], in which pre-programable objects can be fabricated with the ability to change their shape by responding to suitable external stimuli [12].

Herein, a practical and scientific overview of the application of 3D printing technologies in dentistry is provided, specifically emphasizing personalized treatments. First, we discuss the leading 3D printing technologies for manufacturing patient-specific constructs, including selective laser sintering (SLS), selective laser melting (SLM), stereolithography (SLA), digital light processing (DLP), extrusion-based 3D printing, binder jet printing (BJP), and melt electro-writing (MEW). Further discussion is provided regarding the current patient-specific therapeutic devices and constructs for regenerative purposes and the applications of the 3D printing platforms in patient-specific treatment in dental specialties. Then, the last two sections of this review present the concepts and applications of 4D printing in dentistry, the main challenges, and future perspectives for optimizing the techniques and clinical translation of innovative concepts.

## 2. 3D printing technologies for dental applications

Three-dimensional printing techniques have been applied to prepare dental and maxillofacial devices, and their speed, diversity, and applications have continuously advanced. As the classification of 3D printing techniques and their deposition and solidification/curing principles are broadly discussed in the literature, a summary of 3D printing techniques, their advantages and limitations, and their applications for personalized dentistry and maxillofacial surgery are provided (Table 1). This section also includes complementary information regarding these 3D printing techniques discussed throughout this review.

Selective laser sintering and selective laser melting are among the most popular 3D printing techniques for dental applications. SLS and SLM are extensions of the solid freeform manufacturing concept, which uses the thermal energy of a laser to fabricate 3D objects from metal powders or polymers. In SLS, the laser beam is used to sinter the powder at temperatures below its melting point, while in SLM, the temperature rises above the melting point of the powder [13,14]. Another method to form 3D structures from powder beds is binder jetting, also called binder jet printing . However, BJP is a non-beam additive manufacturing technique used on powdered ceramic materials selectively joined using a liquid binder [15].

Other common 3D printing approaches for dentistry are stereolithography and digital light processing, solid freeform-based techniques that apply UV light to cure photosensitive polymeric materials and form 3D pieces. The source and method of application of the UV light are different in these two techniques, which impacts the resolution and precision of 3D printed structures, making SLA more accurate than DLP [16-19]. Also, layer-by-layer addition of the materials has limitations in some applications of 3D printing, for example, printing around a preexisting object. To overcome this limitation, computed axial lithography (CAL) was invented by rotating a photopolymerizable material in a dynamically evolving light field. The technique suits high-viscosity photopolymers and multi-material production [20]. CAL uses a light projector for the polymerization of the resin. This light is applied at seven angles to the materials, producing the entire device in one step instead of layer-by-layer.

Extrusion-based 3D printing, overall, is characterized by the use of pneumatic pressure, pistons, or screw-powered sources to expel the material from a cartridge or syringe [21]. Bioprinting, fused deposition modeling (FDM), and direct ink writing (DIW) are standard extrusion-based 3D printing techniques for dental and craniofacial rehabilitation. Bioprinting employs materials with shear-thinning characteristics to construct layer-by-layer cell-laden scaffolds for tissue regeneration [22]. These materials, so-called bioinks, provide biological cues that mimic the native conditions of the extracellular matrix to favor cell survival in the 3D-printed matrix after printing. Bioinks are ECM-like materials, usually functionalized naturally-based hydrogels (collagen, gelation, chitosan, alginate) or synthetic polymers-reinforced matrices, carrying cells for bioprinting cell-laden constructs [23].

Regarding FDM, most existing machines employ thermoplastic materials, such as polycaprolactone (PCL) and polylactic acid (PLA) etc., in filaments. FDM can print simple anatomical models through fusion between the layers of the molten polymers. However, it still has some limitations regarding the resolution of the printed device when sub-micron scale is intended[24].

Meanwhile, DIW is used to 3D print ceramic materials through a robotic-controlled mechanism of deposition, which makes this technology also known as robocasting [25]. DIW blends a ceramic powder with organic binders to form concentrated, viscous slurries. These composites are then printed at room temperature and maintain the shape of the deposited layers due to their inherent rheological properties. DIW is still the most efficient method for printing ceramic-based constructs due to its versatility, relatively low cost, and minimal need for postprocessing, and its diverse applications, advantages, and limitations for craniofacial tissue regeneration have been recently reviewed elsewhere [26]. Nevertheless, a proper vehicle (e.g., polyvinyl alcohol, co-polymers in other vehicles) should be used to prepare ceramic-based pastes. The adequate balance of the components reduces the brittleness of the paste, avoids clogging the nozzles, and helps to prevent inaccuracy during the deposition and initial lack of stability of the constructs [27].

Melt electrowriting (MEW) is another manufacturing process combining the molten polymer extrusion printing technique and the electrospinning concept, where an electrical field is continuously applied to the extruder. The fiber diameter range goes from ~ 2–50 μm [28] and can reach the nanometric scale field depending on the polymer and the printing parameters [29]. Those sizes are significantly thinner than FDM scaffolds, and the technique presents better control over the deposition when compared to conventional electrospinning, which contributes to guiding cell fate in tissue regeneration strategies [30-34].

Taken together, these 3D printing techniques have allowed significant improvements in building specific dental and craniomaxillofacial devices, and although here we address only those most common currently available processing methods, as the technology continues to evolve, it is expected that other strategies also start to gain some space in the future. Moreover, from an application standpoint, it is necessary to understand the basic concepts of these 3D printing techniques to select the proper materials and their adequate use in dentistry, as further discussed in this review.

## 3. Dental application of 3D printed devices

### 3.1 3D-printed customized oral prostheses

The fabrication technologies of dental prostheses are increasingly becoming automated. So far, subtractive manufacturing methods such as casting and CAD/CAM systems have been applied to produce dental pieces, using a computer numerical control (CNC) machine to remove extra material from a block to obtain desired features [35,36]. Recently, 3D printing emerged to fabricate dental prostheses with fine detail and complex internal geometries from a CAD model [37,38]. 3D printing is expected to offer advantages compared to the traditional fabrication techniques, such as the ability to produce customized dental prostheses with intricate details (e.g., irregular grooves, crannies, valleys), possibly printing various materials simultaneously, reducing production time as well as environmental impact by decreasing energy usage and materials waste [39]. Notably, it facilitates the clinical fabrication of prostheses, as dentists can create, scan, and print patient teeth in a session - saving time and money [40]. An accurate tooth model can be obtained by scanning intraorally or extraorally. The major applications of 3D printing in prosthodontics are the fabrication of fixed prosthetic devices, complete dentures, and removable partial denture frameworks.

#### 3.1.1 Fixed dental prosthesis

Crowns and most bridges are fixed dental prostheses (FDP), cemented onto the existing teeth or implants to repair the damaged teeth. Glass and crystalline ceramics are commonly used for building crowns, veneers, and fixed partial dentures. Lithium disilicate-reinforced glass ceramics, which are commercially available as machinable materials that offer increased strength, toughness, and wear resistance compared to traditional glass-ceramics [41,42], have been commonly processed by subtractive techniques in dental crowns, bridges, and veneers [43]. Due to its advantages, lithium disilicate ceramics have also been 3D-printed via SLA [44] and DIW [45] for dental applications, but these studies are still in the experimental phase. Meanwhile, zirconia-based pure crystalline ceramics (~99% crystalline phase) are prosthetic dentistry’s most common ceramic material. Zirconia is known to have easy machining through CAD/CAM in the pre-sintering stage [46], and it is processed to build crowns, abutments, and even dental implants due to its biocompatibility and osseointegration. 3D-printed zirconia-based prosthetic devices for dental applications have been created via DIW [47]. However, as it occurs for lithium disilicate materials, this model was tested only in vitro with no clinical translation. Therefore, complementary testing steps are necessary for additive manufacturing technologies to print crowns, bridges, and other prosthetic devices from glass and crystalline ceramics.

**Fig. 1.**
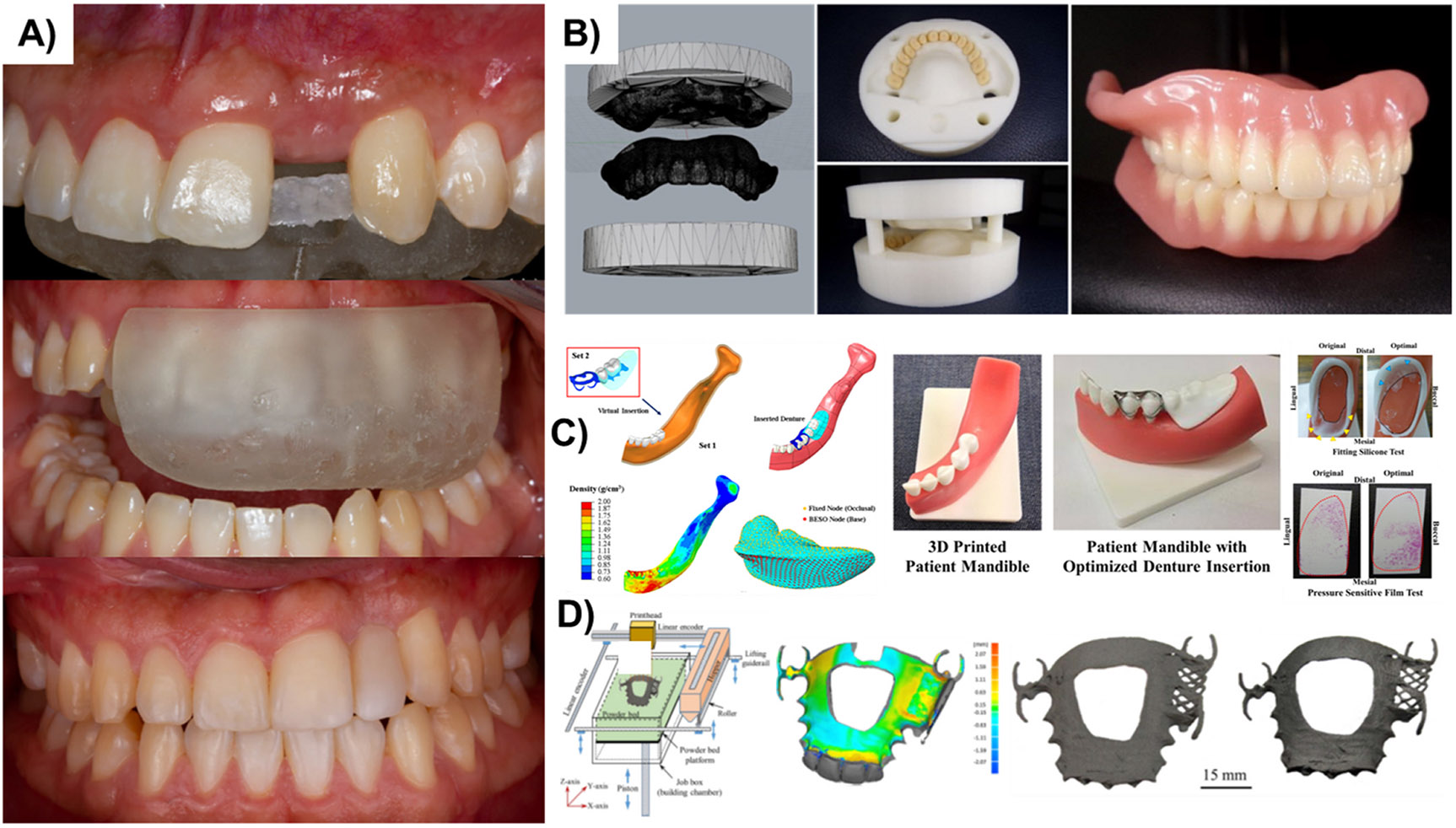
**A)** Utilization of lingual additive manufactured index to guide the fixed dental prosthesis preparation using resin composite in the maxillary front left side (Reprinted/adapted with permission from [52], 2020, Wiley); **B)** The CAD design and 3D-printed upper and lower flasks for optimized fitting of the resin teeth and accurate complete dentures fabrication (Reprinted/adapted with permission from [54], 2020, Elsevier); **C)** CAD model and analysis in vitro and in vivo loading test of the 3D-printed jaw model and the optimized partial denture with a pressure sensitive film (Reprinted/adapted with permission from [55], 2015, PLOS) and; **D)** binder jet printing for the fabrication of RPD metal framework from gas atomized alloy 625 metal powder, and the resulting framework as printed and after sintering (Reprinted/adapted with permission from [56], 2018, Elsevier)

As for the polymeric materials used for fixed prosthodontics, 3D printing methods like SLA or DLP, based on resin materials, can be used for manufacturing crowns and bridges [48,49]. Both interim and fixed dentures should possess proper mechanical, biological, and esthetic properties. They should be able to reduce slight tooth movements, protect periodontal tissues, maintain occlusion function, and guarantee durability and mechanical stability [50]. Compared to interim crowns fabricated using milling or CAD/CAM, those manufactured by 3D printing showed excellent internal fit and edges and greater accuracy [51]. In a clinical study by Gonzalez et al., [52], a digital workflow procedure was defined for replacing an absent maxillary lateral incisor with a 3D-printed FDP using a fiber-reinforced composite. In this report, a unique silicon index was designed using a digital diagnostic wax-up prepared through dental software. As a result, the developed digital workflow improved the digital diagnostic waxing translation into the mouth, providing a controlled insertion route for the index, customizing the location and space of the lingual restoration wings, and diminishing the clinical procedure time (Fig. 1A). Compared with a CAD/CAM milling approach, the fabrication time and wastage of raw materials are reduced using 3D printing techniques, offering a cost-effective option for producing provisional crowns and FDPs [53].

#### 3.1.2. Complete dentures

Traditionally, subtraction technologies, including heat-curing and self-curing methods, have been utilized to manufacture complete dentures. Although polymethyl methacrylate is the most significantly exploited polymer in prosthodontics due to its lightweight, low-cost, simple handling, and stability in the oral environment, polycarbonate is non-biodegradable, with tunable properties, and usually 3D printed into provisional crowns, partial and complete denture bases [57]. Currently, 3D printing can directly fabricate dentures without applying cutting tools, mold, or tooling fixtures [58] and SLS, SLA, and FDM are the most engaged 3D printing technologies in the processing of synthetic polymers used for prosthetic purposes. Conventional complete dentures fabricated via compression molding exhibit higher volumetric and linear shrinkage than 3D printing. Moreover, 3D printing can produce objects with fewer stages, reducing technical errors.

Integrating 3D printing and conventional methods in developing dental prostheses might also be desired. In this regard, Kim et al., [54] used FDM technology to produce customized 3D-printed denture flasks. In their method, conventional dentures were placed into the 3D-printed flasks, packed, finished, and polished using conventional routes to make complete dentures (Fig. 1B). This method showed some advantages compared to the conventional one, where the size of the printed flask was adjustable, which made it possible to produce large-size artificial teeth. No gypsum was required for the 3D-printed flask since the printing materials filled the space around the prosthesis.

Moreover, the use of resin for printing is very cost-effective, with its suitability for rapid prototyping. However, the design and production of artificial teeth using 3D printing are still under investigation [59]. Proper adaptation to the edentulous ridge tissues is essential for masticatory function, removable denture stability, and performance retention [60]. Moreover, manufacturing technologies affect the performance of denture base materials, as 3D-printed prostheses showed superior mechanical properties compared to denture base materials prepared by milling and thermally polymerized acrylic resin [61].

#### 3.1.3. Removable partial denture frameworks

Manufacturing of removable partial denture (RPD) frames has also been optimized by the emergence of 3D printing technologies [62]. 3D printing offers advantages over the traditional waxing process, including the precise fit of the frameworks, reduced mucosal lesions, and less residual ridge resorption. Moreover, the risk of long-term bone resorption is reduced for denture bases produced using 3D printing technology as they can provide more uniform pressure during contact. Recently, clinically usable RDP frameworks have been manufactured using SLM [55]. As Co-Cr alloys are the most common materials to manufacture RPD frameworks, studies have shown that AM technologies reduce the overall porosity of the fabricated Co-Cr alloys, enhancing the printed alloy’s mechanical properties, such as tensile strength [63].

Moreover, it has been reported that Co-Cr alloys produced by SLM displayed higher hardness when compared to conventional fabrication methods [64]. Nevertheless, prosthetic dentistry has also applied polyetheretherketone, a thermoplastic material with high mechanical properties, fracture toughness, and biocompatibility. Polyetheretherketone is a semi-crystalline polymer printed using filaments at high temperatures (~350 °C) to build prosthetic devices, implant abutments, and RPD frameworks [65].

Regarding patient specificity, the automatic design optimization method using finite element (FE) has been integrated with printing technologies to create patient-specific RPDs. Chen et. al, [55] fabricated a patient-specific RPD fitted with quantitative guidelines for correction and adjustment of the denture in a more efficient manner. An FE-prepared 3D heterogeneous model of the mandible area with teeth and the RPD was first fabricated using clinical CT data. 3D printed dentures were tested with pressure-sensitive silicone and films for in vitro investigation of the proposed procedure (Fig. 1C). The fabricated denture showed minimal pain/discomfort, reduced long-term residual ridge resorption, maximum adjustment interval of the denture, exhibiting an evenly distributed contact pressure, and reduced peak pressure (lower than pressure-pain thresholds).

More research has been performed to modify the printing technique and materials to reduce waste and recycling and create more complex geometries. Regarding this, Mostafaei et al., [56] produced an RPD metal framework from gas-atomized alloy 625 metal powder using binder jet printing (Fig. 1D). The sintering stage is crucial in binder jet printing as it affects the final products’ microstructure, dimensions, and porosity. Their results showed a structural density higher than 99% upon applying the sintering process. Moreover, the specimens’ hardness was increased due to sandblasting and mechanical polishing. These mechanical treatments can refine the microstructure’s grains and residual stress of the constructs.

The current advances in 3D printing techniques have provided significant contributions to optimizing the fabrication of both removable and fixed prosthetic devices. Nevertheless, there is still space for progress, mainly on the methods to print ceramic materials in clinically relevant models. Also, technologies to print metal frameworks, such as SLS and SLM, use large equipment that is incompatible with the dental office, which still requests specialized laboratories at higher costs than the conventional methods.

### 3.2. 3D-printed patient-specific implants for maxillofacial surgery and dental applications

Craniofacial defects are a prevalent health issue primarily associated with trauma, congenital conditions, and tumor resection [66]. Treating these lesions or defects includes surgical steps and prosthetic devices to repair or mask the damage while promoting some health condition improvement. Due to the complexity of the craniofacial microarchitecture and the level of impairment that the damage to any tissue could create, maxillofacial surgeries are very challenging and require precise planning and execution. In that regard, advances in imaging devices provide accurate protocols for delicate procedures such as the resection of tumors [67-69]. Combining high-quality images from tomographic scans with specific CAD systems for 3D printing prospects the bioengineering of scaffolds to regenerate critical craniofacial defects. Nowadays, a myriad of medical and research centers work on printing patient-specific implants to repair defects [67,70].

**Fig. 2.**
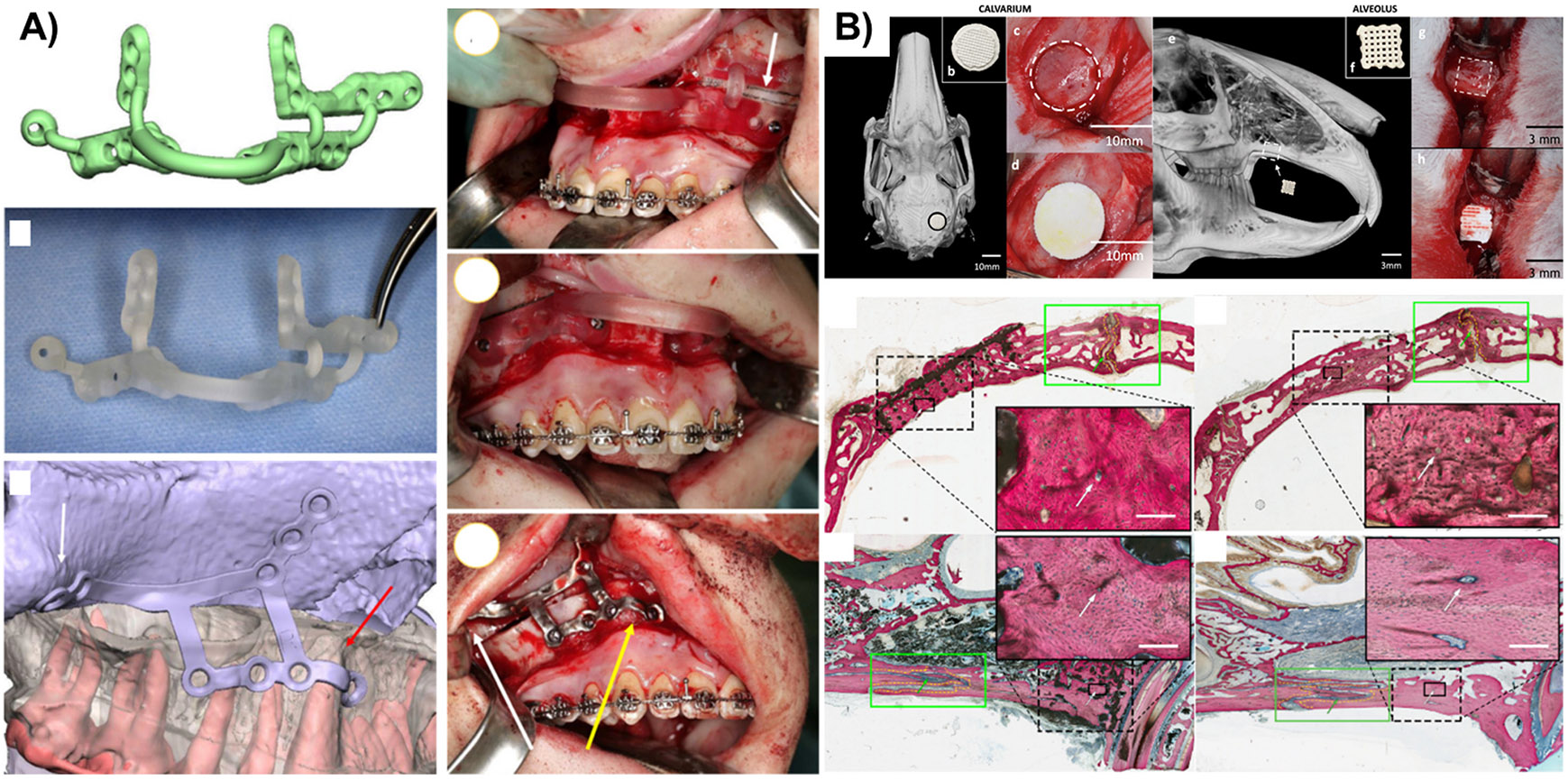
**A)** Site-specific implants and guides based on microtomographies for improving the outcomes of orthognathic surgery(Reprinted/adapted with permission from [67], 2016, Elsevier); **B)** Histological and microtomographic representations of cranial and maxillary defects on immature bone treated with dipyridamole-containing scaffolds. The scaffolds induced regeneration and preserved suture patency. (Reprinted/adapted with permission from [73], 2019, Nature)

Patient-specific implants have indicated a successful combination of cone beam tomography images in multiple areas of maxillofacial surgery, including total joint replacement, reconstruction of maxillofacial disorders, and orthognathic surgery. Regarding this type of clinical need, a freestanding 3D-printed structure based on β-TCP was designed to match the contours of a patient’s face or head, making it ideal for specific defects. A rabbit cranial model has shown that printed scaffolds promote enhanced regeneration of critical-sized bone defects [71]. In a prospective study, twelve patients were treated with orbital wall reconstruction through 3D printed (electron beam melted) patient-specific titanium devices that significantly reduced the surgical time, and, although minor issues still presented concerning the accuracy of the design due to errors in data transferring to STL format, more than 75% of the implants fitted [72]. As the technology was improved, more successful outcomes have been described where a case series reported that pre-bent titanium implants facilitated the implantation of the devices, reduced the clinical time, and presented satisfactory accuracy for Le Fort I and bimaxillary surgeries (Fig. 2A) [67].

Similarly, a randomized clinical trial indicated that patient-specific plates resulted in the precise repositioning of the maxilla in orthognathic surgery [74]. Although these patient-specific 3D-printed devices are an outstanding step for improving the quality of life of individuals affected by craniofacial defects, they are part of a rehabilitation process that is not the optimal treatment for those patients. Therefore, many efforts have been applied in the last twenty years to build constructs that promote complete repair of the anatomical, morphological, and physiological aspects from a molecular basis to the tissue level to regenerate the previously compromised structure.

Most critical defects that demand surgical procedures in the oral and maxillofacial region involve hierarchically organized tissues such as bone, connective tissue, vessels, and nerves. That arrangement is complex to replicate and demands a specific approach to achieve regeneration. Therefore, biofabrication is emerging to build hierarchically arranged and physiologically competent scaffolds to resemble native tissue conditions through additive manufacturing [24]. Those functional constructs aim to replace the need for autologous or non-resorbable synthetic grafts. They can be presented in cell-free or cell-laden strategies to mediate the tissue response and induce regeneration [75]. In both scenarios, many studies address oral and maxillofacial regeneration via 3D printing in different stages of development for further clinical applications.

3D-printed scaffolds for the craniofacial region mainly target critical bone defects and use the FDM printing method and *Food and Drug Administration* (FDA)-approved resorbable polymers (e.g., polycaprolactone, polylactic acid, poly (lactic-co-glycolic acid), polydioxanone, etc.) [24]. Due to the thermoplastic nature of these polymers, FDM is the most utilized technique for their 3D printing applications. Although pristine polymeric constructs are feasible and shape the defects, they lack bioactivity and osteoinductive potential and do not replicate the native conditions of the ECM. To circumvent those issues, functionalized scaffolds containing calcium and phosphate-based materials, drugs, biomolecules, and cells have changed the 3D printing strategies for craniofacial tissue regeneration [76-78,73,79]. 3D-printed PLA scaffolds loaded with extracellular vesicles have demonstrated the ability to induce osteogenic differentiation and bone healing in rat calvaria defects [80]. Also, amorphous magnesium phosphate was mixed with commercially available ECM and 3D printed to promote native conditions for mineralized tissue regeneration. The organic/inorganic phase combination was suitable for cell-free and cell-laden scaffold-based bone regeneration [81].

Besides, extrusion-based 3D printed bioceramic scaffolds offer great cell attachment onto the scaffold surface, even though ceramics are too brittle, impacting the mechanical behavior in load-bearing areas [82]. Therefore, calcium silicate-based BGs have been synthesized with changes in the content of Si or the addition of phosphate, sodium, copper, strontium, magnesium, and other elements to produce composites and improve the properties of these materials [83]. Also, hydroxyapatite (HA), one of the most predominant phases in tooth and bone, and other forms of calcium and phosphate-based materials (e.g., tricalcium phosphates, magnesium phosphate) have been tested to mimic the inorganic phase of bone for regenerative purposes [84]. For instance, site-specific calcium-silicate magnesium-doped (CaSi-Mg) scaffolds were printed to induce bone regeneration in mandibular bone defects. The porous CaSi-Mg scaffolds demonstrated excellent mechanical properties for application in load-bearing areas, high osteogenic potential, and a satisfactory degradability period of up to 16 weeks to regenerate the defects [79].

Moreover, since congenital conditions also result in significant impairment in bone formation, it is also of primary interest to study the tissue regeneration process in different stages of body development. In that regard, dipyridamole-loaded bioceramic constructs were developed and implanted in skeletally immature rabbits to induce bone regeneration [73,85]. The scaffolds demonstrated efficient bone regeneration and degradability ratio and preserved suture patency for calvaria and alveolar defect regeneration (Fig. 2B) [73,85]. This opens new possibilities for performing patient-specific regenerative surgeries in the early stages of bone development to treat palatal clefts, for instance, reducing the risks of ectopic bone formation.

Customization techniques in producing dental and maxillofacial implants may reduce the time for teeth rehabilitation and maintain the soft and hard tissues. Reverse engineering and 3D printing in the production of patient-specific implants eliminate the limitations of conventional machining methods, such as insufficient accuracy and complexity, and enhance the tolerance of implants [7]. Several in vitro, animal, and clinical studies reported the production of 3D-printed titanium (Ti) implants having controlled and adequate porosity levels, along with superficial roughness promoting new bone formation and osseointegration [86]. The main drawbacks in producing 3D-printed implants are surface characteristics, dimensional accuracy, and technology costs [87].

When bone mass around dental implants is inadequate, bone augmentation is one of the existing solutions for successfully inserting implants. Several strategies have been used for bone augmentation in dental implantology to provide stability for individual implants. One uses bone grafting materials in the defect area covered by a Ti mesh. Conventional Ti meshes are required to be manually bent around the bone defect during treatment before being fixed in the site, so the application of customized Ti mesh may result in a shorter surgery time compared to a conventional Ti mesh [88]. Inoue et al., [89] tested customized Ti mesh sheets for the reconstruction of alveolar bone in two cases . A customized Ti mesh sheet produced by SLM was placed simultaneously with the placement of a commercial implant in the first clinical case. The bone morphology was assessed six months after placement using cone beam computed tomography (CBCT) analysis, and the mesh was left in the mouth. In the second clinical case, a similar Ti mesh was applied for augmentation of the bone before the implant’s placement, which was removed after four months of surgery. The process continued with the placement of three commercial implants. Results demonstrated the formation of adequate bone morphology on the postoperative CBCT.

**Fig. 3.**
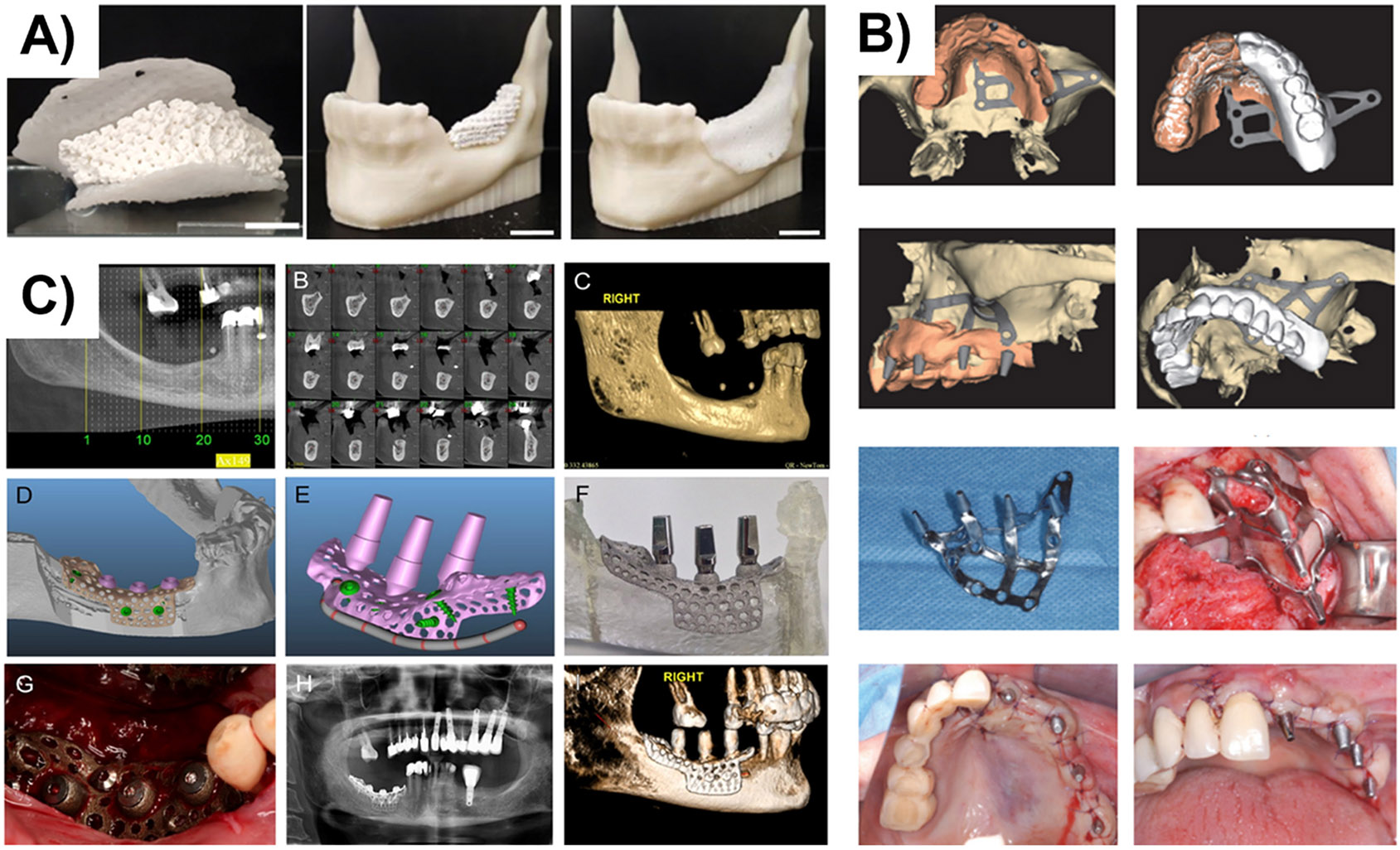
**A)** Assembly of the osteoconductive 3D printed porous TCP/HA fitted into its PCL cover in a model of the mandibular defect (Reprinted/adapted with permission from [90], 2023, Frontiers); **B)** A patient-specific endosteal implant for enhancing bone regeneration (Reprinted/adapted with permission from [91], 2019, Hindawi); C) customized Ti-6Al-4 V implant fabricated as one object and inserted in the patient, a three month follow up using X-ray observation to evaluate osseointegration and the bone to implant contact (Reprinted/adapted with permission from [92], 2016, Nature).

Bone augmentation is also beneficial in extensive oral bone reconstruction. Traditionally, bone augmentation is performed using a manually shaped graft, which requires a long time for fabrication, and its success is often affected by the surgeon’s experience and resources. Therefore, an alternative is needed to customize guided bone regeneration grafts with an exact shape that matches the local patient’s anatomy. In a recent study, the production of personalized osteoconductive devices was reported and proposed for the augmentation of vertical alveolar ridge augmentation by Dairaghi et al., [90]. Porous tricalcium phosphate/hydroxyapatite (TCP/HA) was 3D printed based on a patient-specific model, which was then filled with alginate hydrogel and covered with PCL to anchor and protect the osteoconductive core (Fig. 3A). All the building parts of the model were simultaneously designed in silico from the patients’ 3D images, which were then manufactured using a 3D printer and assembled into one object to be inserted within the originating mandibular defect.

Another predictable method for dental prosthesis rehabilitation in the case of insufficient bone mass is the application of endosseous dental implants, which have a high success rate of long-term survival. A subperiosteal implant is a dental implant inserted between the residual alveolar bone and periosteum. Usually, it comprises two to four transmucosal parts projecting through the mucosa into the oral cavity to connect the prosthesis to the implant [93,94]. Cerea et al., [91] fabricated a custom-made subperiosteal Ti implant using AM to develop an FDP-supporting implant (Fig. 3B). They evaluated the implant-supported FDP based on cemented milled zirconia on a case series of 15 partially edentulous patients in the posterior mandible area. An implant survival rate of 100% and prosthetic complication rate of 30% were reported within the 1-year follow-up. In another similar study, Cohen et al., [92] fabricated a 3D subperiosteal Ti-6Al-4 V bone onlay implant by laser sintering using CT scans of the mandibles of the patients (Fig. 3C). The implants were further surface-modified to create micro/nano textures. The in vitro and in vivo results proved the successful production of 3D-printed patient-specific implants. The surface topography modification of Ti-6Al-4 V implants at the microscale and nanoscale combined with a demineralized bone matrix putty resulted in implant osteointegration and osteogenesis, even for patients with limited bone, resulting in the restoration of bone form and function.

Furthermore, researchers have recently tried to combine specific designs with structural optimization to improve the biomechanical performance of additive-manufactured implants. Pinheiro et al., [95] used structural optimization techniques to develop and numerically validate patient-specific total temporomandibular joint implants. According to their findings, the structural optimization process decreased volume by up to 44% while maintaining the safety and biomechanical effectiveness of the implant.

### 3.3 Personalized 3D-printed scaffolds for periodontal tissue regeneration

Periodontal regeneration can be achieved via different techniques, including guided tissue regeneration (GTR), using either resorbable or non-resorbable membranes [24,96-98], and bone grafting using different bone filling materials or biologics such as enamel matrix derivative (EMD) [99,100]. Even though these techniques yield positive clinical outcomes, periodontal regeneration remains elusive on the human histological level, where periodontal repair instead of regeneration has been reported in animal studies on GTR [101]. Moreover, there needs to be more biological agents suitable for application in all clinical scenarios [99].

**Fig. 4.**
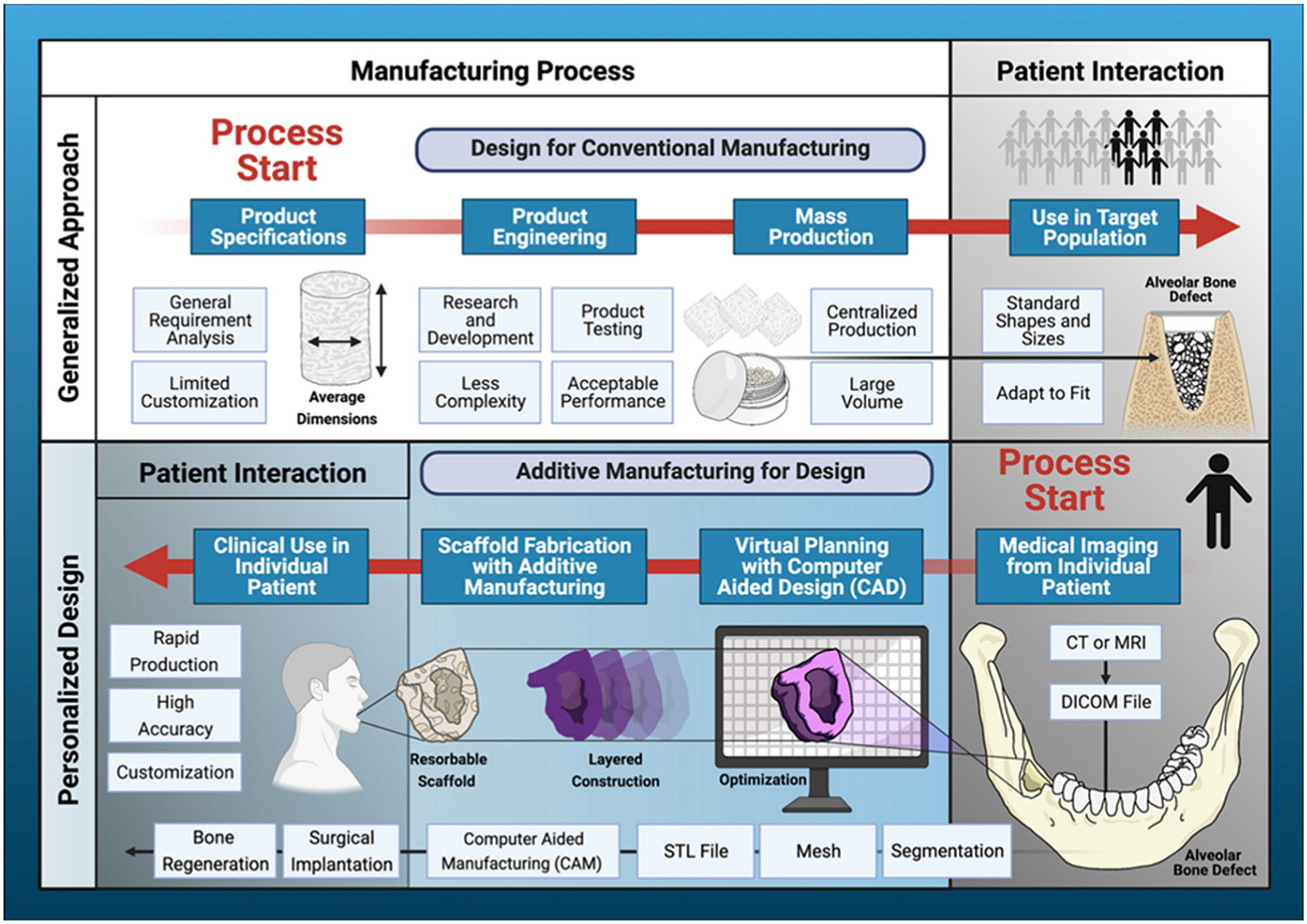
Workflow of designing and manufacturing of custom-made image-based 3D printed scaffolds, in comparison to conventional production methods (Reprinted/adapted with permission from [100], 2021, Frontiers). Traditional generalized method of preparing medical devices using mass production of standard designs and shapes that request adaptations during the implantation to fit the area of interest (top). Personalized devices built from the patients’ CT scans. Conversion to CAD files matching the area, size, and shape of the defects, and subsequent layer-by-layer construction of highly accurate devices using resorbable and bioactive materials with additive manufacturing technologies to induce tissue regeneration (bottom).

In this sense, 3D printing in periodontology started to shift towards personalized therapies throughout the past decade. CAD/CAM technologies are used to print defect-specific scaffolds with distinct geometries to enhance the outcomes of periodontal regeneration. In brief, patient-specific CAD models are generated from CT scans of the defects, allowing 3D printing of the so-called “custom-made” scaffolds that would be applied to regenerate a particular defect [102]. Since those defects are often complex in geometry and the primary goal is to regenerate multiple tissues, combining different manufacturing technologies into a single biofabrication platform has emerged to replicate tissue complexity and functionality [103]. The workflow of designing and manufacturing custom-made image-based 3D printed scaffolds is illustrated in Fig. 4 [100].

Since periodontal regeneration requires spatiotemporal reorganization of alveolar bone, cementum, and periodontal ligament (PDL), multiphasic and compartmentalized 3D printed scaffolds have been introduced in periodontal regeneration. Some previous models have provided important background in that regard by combining functionalized polymeric scaffolds, printed via FDM, with sources of stem cells [104,105], or calcium-phosphate, and growth factors [106] to improve tissue response. Concomitantly, the concept of custom-made image--based 3D printed scaffolds for periodontal regeneration was first reported by Park and colleagues [107,108]. PCL-based scaffolds with “fiber-guiding architecture” of the PDL segment, with a thickness of 250 μm mimicking an adult periodontal ligament width, were designed based on the CT scans of surgically created fenestration defects in rats [108]. The scaffolds were fabricated by casting PCL in a 3D-printed wax mold. Successful in vivo regeneration of bone and obliquely oriented PDL fibers similar to native ligament tissue were achieved, with high periostin expression indicating the PDL matrix’s stability, maturation, and tissue functionality. At the time, casting was an optimal method for scaffold fabrication, but 3D printing technologies have tremendously evolved up to this day.

**Fig. 5.**
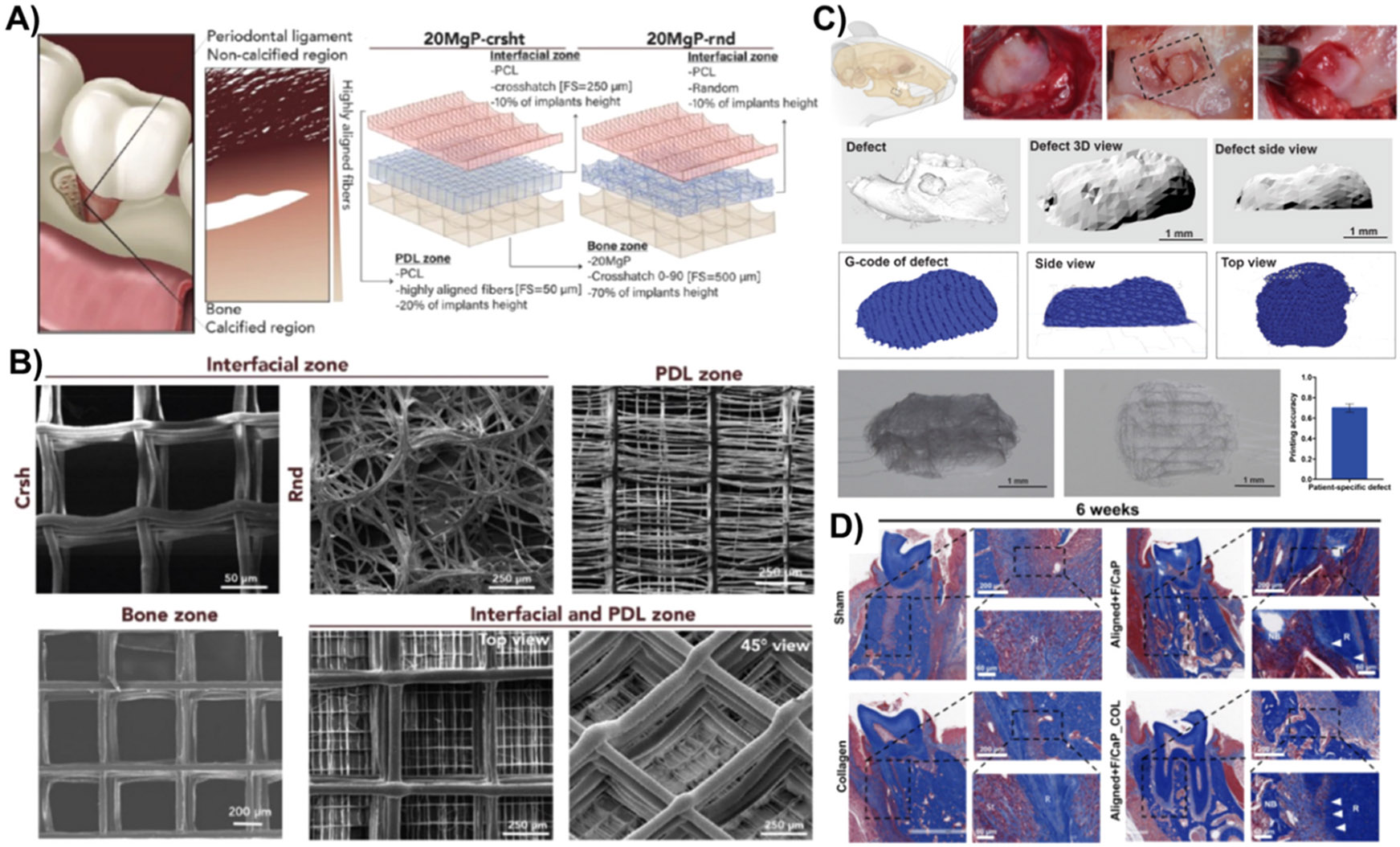
**A)** Representative schematic illustration of the site-specific compartmentalized scaffolds with integrated interfacial zones of the periodontium Reprinted/adapted with permission from [112], 2023, ACS); **B)** SEM images of the characteristic periodontal compartments and transition zones with different fibres’ orientations and strand spaces (Reprinted/adapted with permission from [112], 2023, ACS); **C)** Designing and modelling patient-specific melt electrowritten scaffolds with reliable printing fidelity for periodontal regeneration from CT-Scans of the defects; **D)** and the Masson Trichrome staining of the resulting regeneration in a rat model. [111] (Reprinted/adapted with permission from [111], 2023, Elsevier).

Recent advances in the fabrication of 3D-printed personalized scaffolds have utilized MEW due to its outstanding ability to produce highly defined architectures that replicate the extracellular matrix and micro-environment [109,110]. For instance, Daghrery et al., [111] tested PCL-based highly-oriented 3D-MEW scaffolds with different strand space (250 μm or 500 μm) and morphology of the fibers (aligned or random) to guide human-derived periodontal ligament stem cells differentiation and polarization of macrophage. The aligned fibers’ configuration promoted improved ligamentogenesis and partially downregulated osteogenesis of the stem cells since the scaffold configuration impacts cell commitment. This scaffold was then evaluated on well-established periodontal fenestration defects in rat mandible with reliable printing fidelity from the tomographic reconstructions, which is essential for customization in patient-specific therapies (Fig. 5C-D). The successful regeneration achieved with that model relied on the scaffold architecture, fiber morphology to ensure PDL angular structure at the interface of bone and ligament, and the composition (F/CaP coating). Regarding the latter, it is essential to mention that this was based on the authors’ previous investigation, where they detail the fabrication of this F/CaP melt electrowritten PCL scaffold, which demonstrated antimicrobial features, enabled tissue-specific differentiation of progenitor cells, and resulted in coordinated periodontal regeneration [33].

Recently, another hierarchically organized scaffold was synthesized via MEW in a single-step printing [112]. Compartmentalized PCL-based MgP-containing scaffolds presenting bone zone, PDL zone, and a transitional interface were characterized and tested in vivo on periodontal fenestration defects (Fig. 5A-B). Incorporating magnesium phosphate into the scaffolds significantly increased the expression of bone markers and favored stem cell differentiation towards bone regeneration, while the aligned PCL fibers contributed to regenerating PDL. Regarding the in vivo model, a random architecture in the interfacial zone, connecting PDL and bone, significantly improved the hierarchical organization and coordinated transition of the periodontal tissue compartments.

**Fig. 6.**
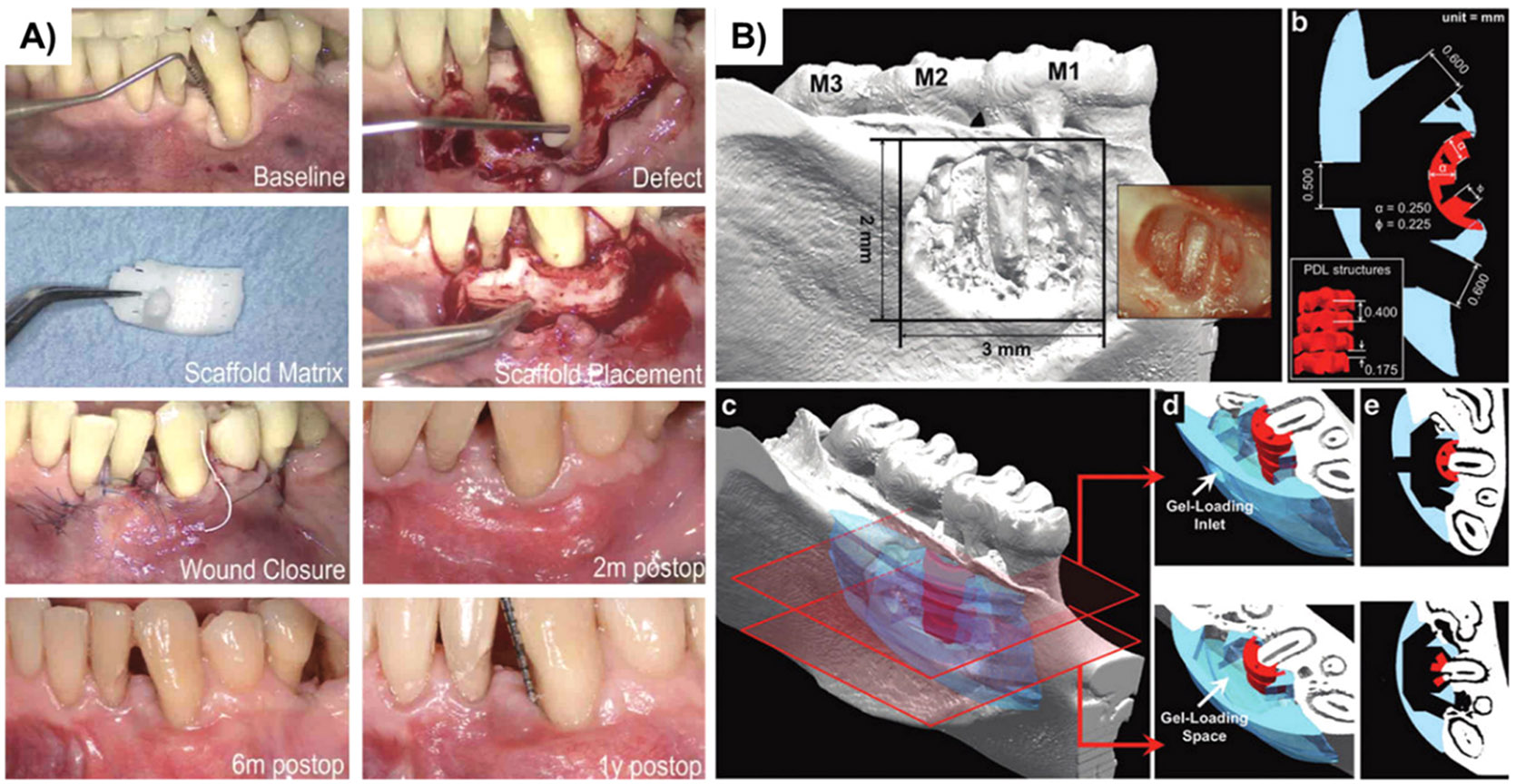
Custom-made image-based 3D printed scaffold for periodontal regeneration. **A)** Case Report of the implanted image-based 3D printed PCL construct for periodontal regeneration (Reprinted/adapted with permission from [113], 2015, SAGE); **B)** Imaging and design of the 3D CAD model for in vivo regeneration in a fenestration defect in the rat mandible (Reprinted/adapted with permission from [108], 2012, Elsevier).

From a clinical standpoint, a single case report is available in the literature, where a PCL-based scaffold was designed from a patient’s CT scan to regenerate a complex periodontal defect in the anterior mandibular area (Fig. 6A) [113]. The scaffold was printed using SLS, with some enclosed grooves to allow PDGF infusion, and demonstrated a high adaptability ratio (~96%) to the defect. After 13 months of the scaffold insertion, soft tissue dehiscence was evident with subsequent scaffold expulsion, attributed to the slow degradation rate of PCL. Interestingly, histological analysis showed that 76% of the total molecular weight of the scaffold remained in the defect and was not remodeled, which elicited soft tissue dehiscence and eventual scaffold failure. Therefore, modifying the degradation rate of PCL or using composite scaffolds made of PCL and another polymer with a faster degradation rate is highly recommended for periodontal tissue regeneration purposes. Although the outcome was relatively unsuccessful, it must be noted that this model demonstrated promising results in animal studies (Fig. 6B) [107,108,114]. Still, the inherent differences between humans and rats in terms of host responses, anatomic factors, and healing window probably influenced the predictability of the clinical result.

To summarize, advances in periodontal regeneration aim to regenerate multiple lost tissues that are fully functional. 3D-printed scaffolds, using various additive manufacturing technologies, have been widely tested in the regeneration of periodontal tissue, both in vitro and in vivo, with successful outcomes. However, due to the limitations of individual 3D printing technologies in capturing the complexity of periodontal tissue, the convergence of 3D printing techniques into a single platform is the possible path to pave the future clinical translation of periodontal tissue regeneration.

### 3.4. 3D printed customized orthodontic devices

3D printing has revolutionized orthodontics practice in recent years by influencing the diagnosis, orthodontic treatment plan, and process [115,116]. As a result, a wide variety of customized orthodontic appliances, including brackets, archwires, and aligners, have been developed rapidly.

**Fig. 7.**
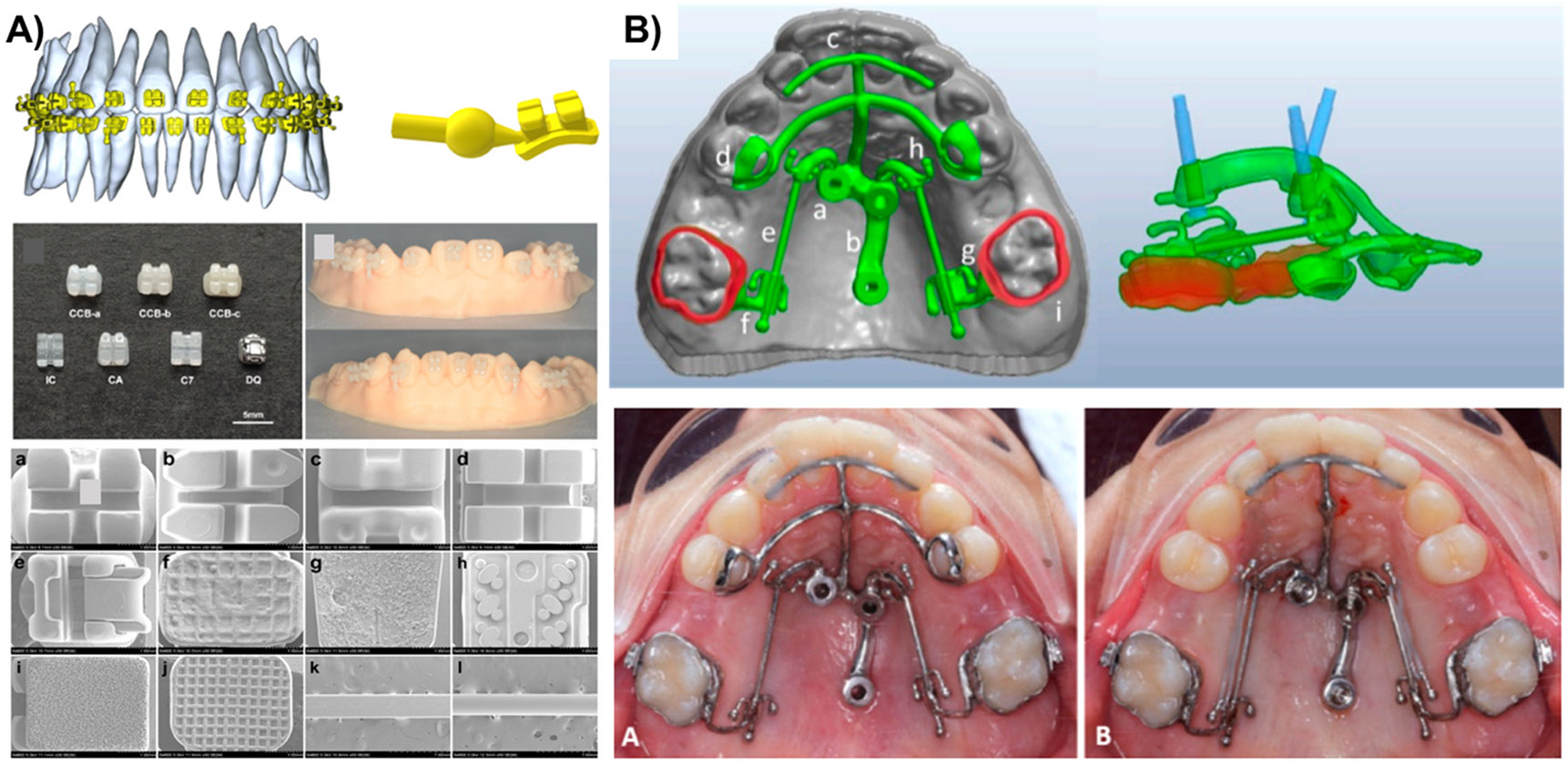
**A)** Development of a customized esthetic ceramic orthodontic bracket system, which employed the individual digital design, lithium disilicate materials, and heat-pressing technology (Reprinted/adapted with permission from [117], 2019, Springer; **B)** 3D design of DIVA device from the 3D design showing the part of the appliance to its final placement in the oral cavity (Reprinted/adapted with permission from [120], 2021, MDPI)

For instance, Yang et al., [117] have developed a standard manufacturing technique based on individual digital design and heat-pressing treatment to produce customized esthetic ceramic brackets from lithium disilicate materials (Fig. 7A). Recently, in a clinical study by Kara-boulad et al., [118], the HIRO system was modified with a 3D printer to three-dimensionally align the teeth into their desired places and to fabricate the printed final model on which lingual brackets were placed. Some advantages of that method included accurate placement for each bracket without being affected by operator mistakes, proper control of the width and length of the dental arch at the end of the process, minimum laboratory equipment, and cost-effectiveness. Apart from that, an essential part of quality in orthodontic treatment is the standard production of archwires. Rapid development in 3D printing technology has led to the design and production of personalized archwire models [119], which have demonstrated uniform distribution of dentofacial stress, improved patient wearing comfort, and decreased tooth repair and treatment time.

Regarding more recent techniques, clear aligners are new orthodontic devices made of colorless and transparent thermoplastic polymers. Researchers hope to develop novel aligner materials, particularly for application in direct 3D printing. Jindal et al., [121] reported successfully producing a 3D-printed 0.75-mm thick clear aligner from Dental LT^®^ clear resin. The 3D-printed resin presented accurate geometry, higher resistance to maximum load with a low displacement, and the ability to deform elastically with reversibility for lower displacements than conventional aligners. A recent animal and clinical study used a combination of FE and 3D printing to fabricate a personalized study model for manufacturing clear aligners [122]. Both animal experiments and clinical cases exhibited the feasibility of the ‘invisible’ orthodontics by polyurethane clear aligners.

Furthermore, 3D printing offers more accuracy in manufacturing occlusal splints as the best fit for the patient. One advantage of 3D printing is the possibility of customized splints quickly utilizing modern digital technology, reducing costs and clinical working time. Eruption Guidance Appliances (EGAs) is an orthodontic device recommended for early orthodontic treatment to correct sagittal and vertical occlusal relations as well as alignment of the incisors. Barone et al., [123] designed patient-specific EGAs using CAD modeling software and tomographic data from a CBCT scan. The customized EGA effectively reduced the stress level inconsistency in condyle disks. In addition, the developed customized EGA could be applied to treat other malocclusion disorders, such as misalignments of individual teeth within dental arches.

In orthodontics, anchorage methods like mini-screws and mini-plates can treat disorders such as molar intrusion or distalization, open bite correction, and maxillary impaction or protraction [124]. The production of surgical guides using 3D printing technologies is increasingly becoming popular since it provides a simple and safe method of inserting mini-implants, with customized adaption, precision, and accuracy for both miniplate and mini-screw placements, maximum surface contact between miniplate/mini-screw and bone, and lower failure rates of mini-screws [125,126]. Regarding this, Cantarella et al., [120] customized an orthodontic appliance on the morphology of maxillary bone using patient CBCT data. This miniscrew-supported Divergent Anchor device was designed and fabricated using SLM (Fig. 7B). The patient-specific appliance was cemented first and applied as a surgical guide for placing mini-screws for sagittal or vertical orthodontic tooth movement.

3D printing can also be utilized to manufacture study models for orthodontic applications due to its visualization, accuracy, and accessibility [127,128]. The 3D-printed models can be used to produce removable orthodontic devices, indirect bonding trays, expansion appliances, and thermoplastic aligners [129]. The anatomical models fabricated using 3D printing from CT images provide thorough visualization and anatomy assessment for the clinician of impacted tooth localization and surgical exposure procedures for the impacted tooth [130]. Similarly, 3D printing offers an alternative in orthodontics to manufacture customized removable retainers. In the first step, CBCT imaging is used to create a 3D model of the patient’s dentition. After importing the 3D model into a dedicated software program, the retainer is virtually designed and 3D printed, and finally, a clear plastic retainer can be directly produced [131].

As orthodontics naturally involves patient specificity and individualized treatments, it is natural to expect that the currently available 3D printing technologies combined with images from CT scans are permeating the clinical appliances and optimizing the quality of the proposed features.

### 3.5. 3D printing in endodontics

Traditional root canal therapy consists of chemical-mechanical removal of the infected tissue and sealing the canal with gutta-percha before coronal filling with a restorative material. Nonetheless, many studies have resorted to specific biologically driven strategies looking for better outcomes while dealing with injuries to the dental pulp, either in an attempt to preserve tooth vitality or to induce dental pulp regeneration. Several studies investigated drug-loaded scaffolds for the controlled release of antibiotics and chemical agents in the root canal system to preserve the regenerative capacity of the tissue [132-136]. However, 3D-printed scaffolds accurately reproducing the complex anatomy of the root canal or clinically relevant models are still to be explored for predictable and successful responses.

To date, most studies addressing the pulp-dentin complex regeneration have not used anatomically shaped 3D-printed constructs. However, functionalized 3D matrices have been tested to induce regeneration of that region [137-139]. Concerning the 3D printing approaches, a combined alginate and dentin matrix-derived bioink was formulated to print suitable constructs to induce odontogenic differentiation and regenerate the dental tissue [140]. The alginate-dentin matrix bioink demonstrated satisfactory printability parameters and improved the SCAPs’ ability to differentiate into odontoblasts [140]. Notably, tubular electrospun constructs can be designed to fit the canal shape, creating patient-specific scaffolds for endodontic disinfection. However, some limitations remain regarding hard-to-access regions such as accessory or atresic canals. Unless the clinician enlarges the root canal during mechanical preparation, developing 3D-printed scaffolds to fit the canal in those situations is challenging for pulp tissue engineering. For those scenarios, injectable and photo-curable functionalized hydrogels are a more suitable alternative for clinical applications [136,141].

**Fig. 8.**
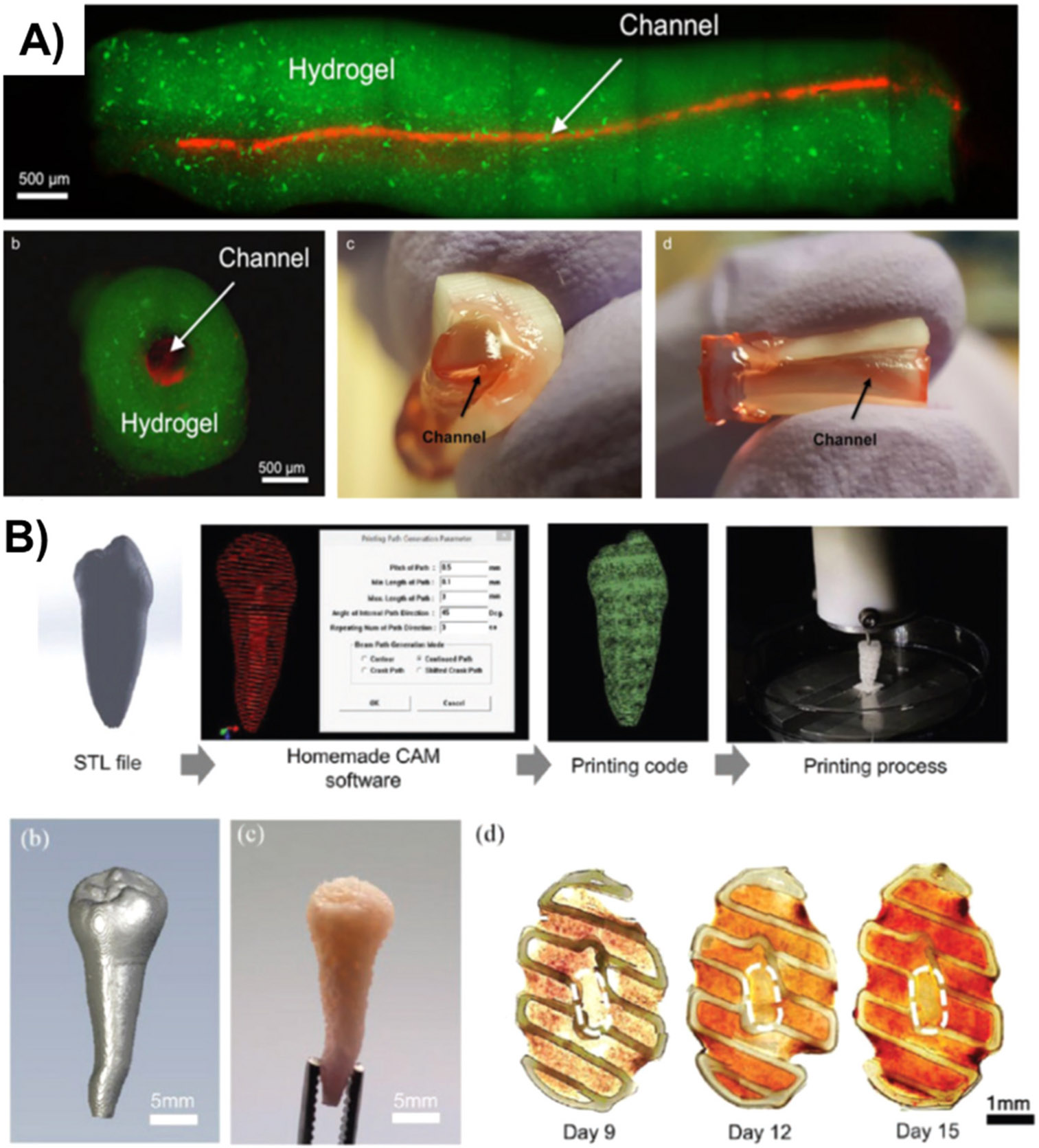
**A)** Cell-laden hydrogels containing stem cells for a full-length regeneration of the pulp tissue. (Reprinted/adapted with permission from [142], 2017, Nature); **B)** 3D printed model of a whole dentin-pulp complex reconstruction (Reprinted/adapted with permission from [143], 2019, Sage)

The final bioengineering goal for the dental pulp is to achieve complete regeneration of the loose connective tissue and its vascularization and innervation. A complex system of progenitor cells and growth factors is necessary to restore pulp vitality after root canal disinfection. Therefore, combining stem-cell transplantation and biomolecules within 3D printed constructs in bioprinting techniques is an emerging field for designing new pulp tissue. It has been demonstrated that cross-linkable hydrogels are an attractive biomaterial as support for carrying those cells in bioprinting. For instance, a full-length pre-vascularized pulp-like tissue was prepared using co-cultures of undifferentiated pulp cells (OD21) and endothelial cells to combine the cells with the potential for odontoblast differentiation with revascularization through prefabricated microchannels [142] (Fig. 8A). The stiffness of the gels influenced cell spreading and proliferation, and the OD21 was prone to move toward the dentin walls. In contrast, the endothelial cells formed endothelial monolayers for the first steps in vessel organization [142].

Likewise, fibrin-based bioinks were prepared for spatially organized 3D bioprinting of DPSC for the whole pulp-dentin complex regeneration [143]. By using 3D-printed PCL for the outer shape of the tooth and the hDPSC-laden bioinks to rebuild the pulp-dentin complex, the authors could print the constructs from converted microtomography images. The concentration of the fibrinogen hydrogel did not affect the cell viability and allowed proliferation with a dentin-like pore size at the highest concentration of fibrinogen [143] (Fig. 8B). Apparently, by controlling the stiffness, it is possible to adjust the outer layer to function as the hard tissue and the inner layer to guide the soft tissue regeneration, which supports the hypothesis of using 3D printing and bioprinting for the regeneration of the pulp-dentin complex in a single patient-specific model.

For future perspectives, the convergence of technologies could be relevant to addressing the design of compartmentalized constructs that modulate cell fate and differentiation to fully regenerate the pulp tissue vascularization and innervation and guide the mineralized tissue formation for the outer dentin layers. Combining those approaches with high-resolution microtomography images would also help to understand the root canals’ complex anatomy and progress toward using 3D printing technologies to regenerate the dental pulp in the clinic.

### 4. 4D printing for personalized dental treatment

4D printing is an advance in 3D printing encompassing stimuli-responsive materials that can exhibit a pre-programmed function. The term was introduced by Skylar Tibbets in his 2013 Ted talk [144]. It adds time as a new dimension to the 3D constructs that transform functionally or morphologically over time as desired in response to a stimulus [145]. The materials employed for 4D printing are auto-repair, auto-sensing, auto-responsive, and auto-adaptable, and must possess multi-functionality. These materials are mainly described as presenting shape-memory and shape-changing features. Shape-memory materials go into a fixed temporary shape, remaining stable but returning to the original when a stimulus is applied. On the other hand, shape-changing materials maintain their original form but go into a temporary one when a stimulus is applied [146]. Since its emergence, there has been a growing interest in exploring 4D printing in the design of biomedical devices [147]. 4D printing is an up-and-coming tool for developing biomedical devices such as those that can be autonomously deployed; one such example is the fabrication of a 4D shape-memory airway stent. The stent is delivered in its temporary shape and, due to the shape-memory effect, is triggered to its original permanent shape to match the anatomy of the target [148].

3D printing has successfully provided personalized designs for medical implants, and as the quality of materials improves, so does that of the implants [149]. 4D printing, on the other hand, provides not just personalization but high precision for printing 3D constructs to mimic natural tissues. A polymer hydrogel that undergoes shape-memory effect has been used to develop soft-tissue implants that may find use in orthopedics. Other 4D-printed shape memory polymers (SMPs) can also be used to design medical scaffolds including bone scaffolds [149]. 4D printing may also find use in prosthodontics as crown copings, bridge pontics, and frameworks for partial dentures. Generally, while the hard and soft tissues around pontics are changing, the seating of the latter may remain poor. SMPs may be used instead of pontics; thus, their self-adjusting capability may prevent complications. SMPs could also be used in denture re-lining to adapt to the surrounding altering hard and soft tissues [57]. Advanced denture bases manufactured using 4D printing with similar elasticity and thermal features as the oral tissue may be adapted to the different types of applied stress in the oral cavity [150,151].

Since provisional crowns in prosthodontics are vital for the health of soft tissues, they suffer from the problem of improper fitting. A patent was filed for a shape-memory material (SMM) resin that fits the tooth abutment and restores it provisionally without cement and, on temperature change, it fits the abutment firmly [152]. SMMs have been used in dentistry for a long time. For instance, Ni-Ti orthodontic archwires were first used in 1971 [153]. SMMs can also evoke a cellular response against a mechanical stimulus in orthodontic tooth movement. Smart and removable orthodontic devices such as arch extension and bite-raising can also be 4D printed.

In implantology and maxillofacial surgery, implants, drug mouth-guards, and surgical guides are possible examples of 4D-printed devices. Regarding this, titanium implants might be replaced with shape memory materials to enhance implant biocompatibility and osteointegration. Shape-memory alloys may find use in endodontics. They may alter according to the curvature of the root canal, preventing any reinfections or biofilm formation and thus replacing traditional instruments. Similarly, advanced filling materials might be applied in inaccessible parts of the oral cavity, adaptable to predictable movement, and helpful in resolving current complications, including polymerization shrinkage, dimensional changes, and microleakage. SMPs may also be used in implantology and might replace titanium and its alloys since they have more tunable properties, are more biocompatible and biodegradable, have flexible programming, can be deformed elastically, prevent microleakages, and cause expansion and contraction like natural teeth [57,153].

Though 4D printing is still in its infancy, and there are many bottlenecks to be addressed, where the most imperative one seems to be the materials, the fabrication of materials that respond to diverse stimuli and can produce complex structures is probably a viable solution to optimize 4D printing for future applications in dentistry.

## 5. Challenges and future perspectives

In the past decade, there have been rapid advances in 3D printing technology across various medical fields, including dentistry. The unique features of 3D printing, including ease of production, short fabrication time, ability to customize, and material versatility, make it an attractive manufacturing technique for dental devices. The low material waste of 3D printing compared to traditional manufacturing methods is another advantage of the process. Developing new feed materials and printing methods may speed up fabrication.

Despite all benefits, additive manufacturing still faces several challenges, such as controlling design parameters, the efficiency of the devices, printed material biocompatibility, and sterilization. Additionally, materials availability, medical features of the materials, and required printing resolution and time should be considered when selecting suitable printing systems. Practically, printed materials are weaker than traditionally used artificial tooth materials and denture base resin [154]. In addition, compared to milling, 3D printing materials are more challenging to develop [155]. After designing a suitable material system, significant effort and time will be needed before it is approved for clinical use. Another challenge is the properties of the cell-laden bioink; the fragility of printed objects made of cells and the complexity of manufactured structures require a carefully planned procedure.

From a regulatory perspective, the printing materials must meet the required standards for dental application in terms of bioactivity and technical aspects. Thus, developing new printable dental materials that meet these specifications will be of great interest, as new opportunities for 3D printing in dentistry are also created by expanding the material range. Besides the properties of applied materials, it is required to carefully consider the following items: 3D printer running, maintenance, and materials-related costs, as well as the need for skilled operators, post-processing, and adherence to strict health and safety protocols. While significant advances have been made in many of these areas, additional studies are required before implementing effective therapies. Introducing 3D-printed goods to the market and their wide adoption in the healthcare sector, specifically for highly regulated markets, is another challenge that 3D printing faces as a new manufacturing technology. By implementing 3D printing, benefits for patients and the healthcare system may be achieved, making the amount of research required to establish a process for manufacturing customized products reasonable.

In the future, the high demand for customization and patient-specific devices may require dentists and surgeons to have manufacturing apparatus in their offices or hospitals. Patient-specific therapy in dentistry and maxillofacial surgery is gradually becoming a reality in several leading hospitals worldwide, which brings together multidisciplinary researchers and technicians like computer scientists, biomaterials engineers, and dentists. Applying advanced materials and producing 4D-printed constructs is the next-generation technique for fabricating transformable structures in biomedicine. The future of digital dental treatment and maxillofacial rehabilitation may be revolutionized by 4D printing. Fixed and removable devices produced by 4D printing may enhance treatment efficiency and strengthen the digital process in addition to diagnostic and imaging tools.

## 6. Conclusions

3D printing makes it possible to produce accurately complex objects from digital data using various materials in local centers or industries. Its application in dentistry and maxillofacial surgery has led to significant advancements in personalized treatments and the fabrication of patient-specific constructs. Various 3D printing techniques, such as selective laser sintering, stereolithography, digital light processing, extrusion-based printing, and melt electrowriting, have been employed to create dental and maxillofacial devices with improved speed, diversity, and applications. The use of 3D printing in dentistry encompasses various areas such as prosthodontics, periodontics, orthodontics, endodontics, and maxillofacial surgery. The technology has enabled the production of patient-specific dental prostheses, including fixed dental prostheses, complete dentures, and removable partial denture frameworks. The use of 3D printing in orthodontics has allowed for the development of customized esthetic ceramic brackets, archwires, and clear aligners. In periodontal tissue regeneration, the use of multiphasic and compartmentalized 3D-printed scaffolds has been introduced to replicate tissue complexity and functionality, particularly for the spatiotemporal reorganization of alveolar bone, cementum, and PDL. 3D printing also enables the bioengineering of scaffolds to regenerate critical craniofacial defects as well as the production of patient-specific implants in various areas of maxillofacial surgery, including orbital wall reconstruction, orthognathic surgery, and bone augmentation with improved surgical outcomes, reduced surgical time, and promoted bone regeneration. Additionally, 3D printing has shown promise in endodontics for developing biologically driven strategies for dental pulp regeneration. 4D printing as an advanced AM technology represents a significant progress in dental treatment, offering stimuli-responsive materials that can exhibit pre-programmed functions. The use of 4D printing in dentistry holds promise for creating personalized and high-precision 3D constructs that mimic natural tissues. Additionally, the widespread adoption of 3D printing and 4D printing in dental care may require interdisciplinary collaboration and the establishment of new manufacturing processes to meet the demand for patient-specific devices.

## Figures and Tables

**Table 1:** Different 3D for dental and maxillofacial and limitations.

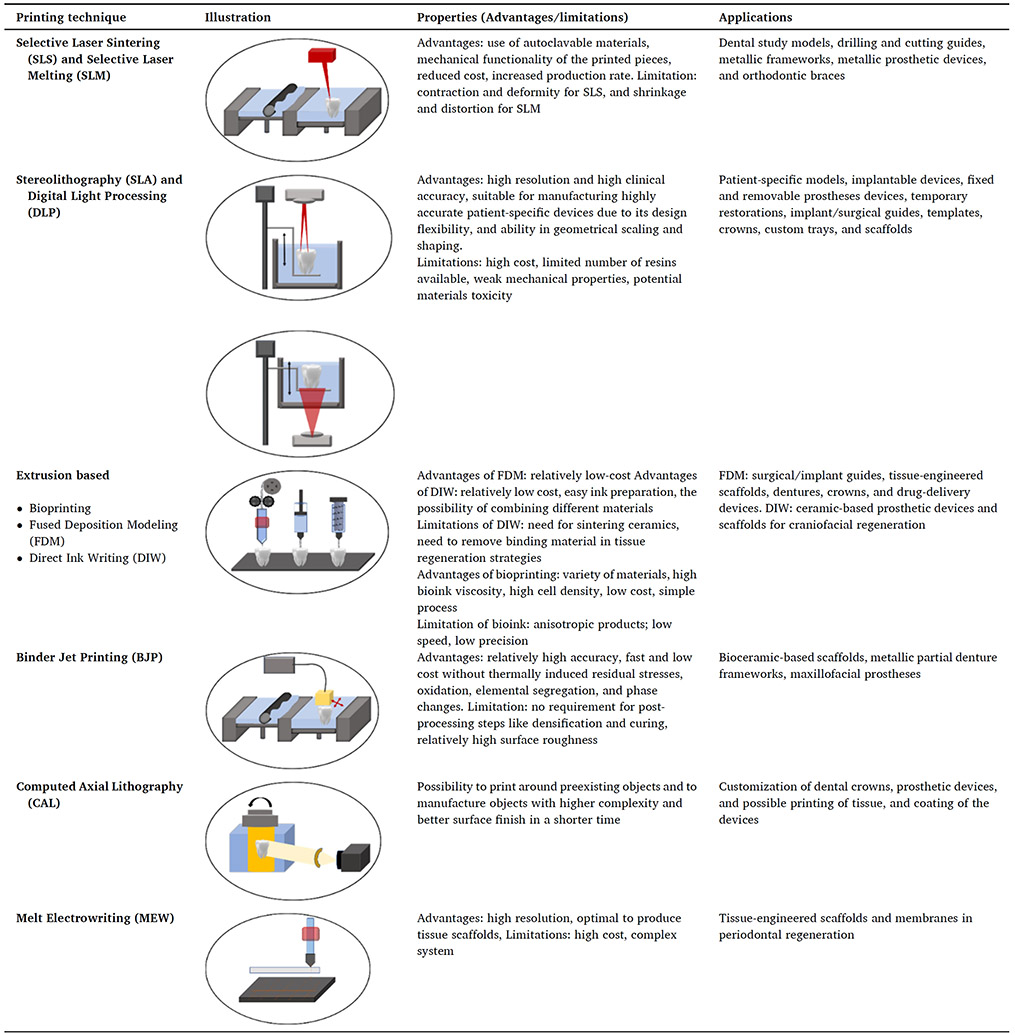

## Nomenclature

### Materials

EMD: Enamel matrix derivative
HA: Hydroxyapatite
PCL: Polycaprolactone
PLA: Polylactice acid
PDL: Periodontal ligament
SMM: Shape memory materials
SMP: Shape memory polymer
TCP: Tricalcium phosphate
Ti: Titanium

### Techniques

AM: Additive manufacturing
BJP: Binder jet printing
CAD: Computer aided design
CAL: Computed axial lithography
CBCT: Cone beam computed tomography
CNC: Computer numerical control
DIW: Direct inkjet writing
DLP: Digital light processing
EGA: Eruption guidance alliance
FDA: Food and drug administration
FDM: Fused deposition modeling
FE: Finite element
GTR: Guided tissue regeneration
MEW: Melt electrowriting
SLA: Stereolithography
SLM: Selective laser melting
SLS: Selective lase sintering
STL: Stereolithography (file format)

### Structures

3D: Three-dimensional
4D: Four-dimensional
FDP: Fixed dental prostheses
RPD: Removable partial denture
